# LAMP Coupled CRISPR-Cas12a Module for Rapid, Sensitive and Visual Detection of Porcine Circovirus 2

**DOI:** 10.3390/ani12182413

**Published:** 2022-09-14

**Authors:** Lei Lei, Fan Liao, Lei Tan, Deyong Duan, Yang Zhan, Naidong Wang, Yuge Wang, Xiaoye Peng, Kaixin Wang, Xiaojiu Huang, Yi Yang, Aibing Wang

**Affiliations:** 1 Hunan Provincial Key Laboratory of Protein Engineering in Animal Vaccines, College of Veterinary Medicine, Hunan Agricultural University (HUNAU), Changsha 410128, China; 2 PCB Biotechnology LLC., Rockville, MD 20852, USA

**Keywords:** porcine circovirus 2, CRISPR/Cas12a, crRNA, LAMP, visual detection, qPCR

## Abstract

**Simple Summary:**

In this study, we aimed to establish a visual, rapid, low-cost, sensitive, specific, and portable nucleic acid detection method for PCV2 through coupling LAMP with CRISPR/Cas12a. All the results of LAMP-CRISPR detection, including a low detectable limit of 1 copy/μL, no cross-reaction with main porcine DNA or RNA viruses, and a 100% coincidence rate with qPCR detection, demonstrated that this method was reliable. It has laid the foundation for developing a PCV2 detection kit based on this LAMP-CRISPR method.

**Abstract:**

Porcine circovirus 2 (PCV2) is the main pathogen of porcine circovirus-associated disease (PCVAD), which can cause considerable economic loss to the pig industry. The diagnosis of PCVAD is complicated and requires a series of clinical, pathological, and virological methods. Therefore, a rapid, highly sensitive, on-site, and visual diagnostic approach would facilitate dealing with the spread of PCV2. In this study, we intended to establish a new and effective PCV2 detection method through combining the no specific equipment requirement advantage of loop-mediated isothermal amplification (LAMP) with the property of clustered regular interspaced short palindromic repeats (CRISPR)/Cas12a system possessing the huLbCas12a collateral cleavage activity able to cleave single-stranded DNA fluorophore quencher probe sensor (designed as LAPM-CRISPR). Following a series of optimizations of its reaction conditions, this LAMP-CRISPR-based PCV2 detection could be conducted in constant temperature equipment, with the result reflected in a direct visual readout way. This established PCV2 detection approach presented fine sensitivity, rapidity, specificity, and reliability, as demonstrated by a low detectable limit of 1 copy/μL, completed within an hour, no cross-reaction with main porcine DNA or RNA viruses like PCV1, PCV3, and PEDV, and a 100% coincidence rate with that of the quantitative PCR (qPCR) method in the evaluation of 30 clinical blood samples, respectively. Therefore, this novel method makes rapid, on-site, visual, highly sensitive, and specific detection of PCV2 possible, facilitating the prevention of this pathogen in the field.

## 1. Introduction

Porcine circovirus is one of the smallest animal viruses ever discovered. Porcine circovirus 2 (PCV2) was first reported in Canada in 1991, which was isolated from sick pigs with diarrhea, central nervous system disorders, and symptomatic disease in vivo [1]. The virus preferentially attacks the lymphoid tissue of pigs, leading to lymphoid tissue failure and immunosuppression [2,3]. PCV2 is also considered the main pathogen causing porcine circovirus-associated disease (PCVAD), with the consequences of immunosuppression, porcine dermatitis and nephrotic syndrome (PDNS), proliferative necrotizing pneumonia (PNP), porcine respiratory disease complex (PRDC), reproductive disorders, congenital tremors, enteritis, and other diseases [4], thereby leading to serious economic losses in the pig industry. PCV2 is a single-stranded circular DNA virus with a genome size of ~1760 bp, belonging to the circovirus family [5]. The genome of PCV2 contains eleven predicted open reading frames (ORFs) that encode replicase (Rep), capsid (Cap), apoptosis-mediated proteins, and other functional proteins [6]. PCV2 is considered a DNA virus with a high mutation rate, which leads to the emergence of a large number of variants over time [7]. According to different typing criteria, PCV2 is divided into different genotypes [8]. The most accepted protocol classifies PCV2 into eight genotypes (PCV2a to PCV2h) [9]. Furthermore, a new genotype, designated as PCV2i, was reported in the USA [10]. Notably, three genotypes, including PCV2a, PCV2b, and PCV2d, display global distribution currently [9]. For instance, in 2020, Gou Z et al. analyzed 76 enteric samples from piglets with severe diarrhea disease by qPCR in Henan, China, and discovered that two PCV2 genotypes, PCV2b and PCV2d, were circulating in the fields [11]. Similarly, Li et al. collected and analyzed the sequences of 714 PCV2 strains submitted to the GenBank database in China from 2000 to 2019, and found that the prevalence of PCV2b and PCV2d strains was dominant over time [12]. Importantly, the widespread of different PCV2 strains, together with the high prevalence of multiple swine virus co-infections, poses a serious threat to the development of the swine industry.

To date, there are many specific PCV2 detection methods, which are mainly divided into two diagnostic techniques for probing the presence of nucleic acids or serum antibodies of this pathogen, including virus isolation, electron microscopy, polymerase chain reaction (PCR), real-time fluorescence quantitative PCR (qPCR), fluorescence in situ hybridization (FISH), loop-mediated isothermal amplification (LAMP), immunofluorescence assay (IFA), immunohistochemistry (IHC), and enzyme-linked immunosorbent assay (ELISA) [2,13,14,15,16,17,18,19]. Although those diagnostic methods have widely been applied in the detection of viruses and antibodies, they require expensive and special instruments, professional personnel, and complex procedures. These issues make real-time detection in the field almost impossible. Hence, a highly sensitive, rapid, and visual diagnostic method is essential for PCV2 point-of-care detection.

Recently, clustered regular interspaced short palindromic repeats (CRISPR) and a CRISPR-associated system (Cas) containing programmable endonucleases can be repurposed for CRISPR-based diagnostics. In particular, the developed detection methods based on the trans-cleavage activities of Cas12 and Cas13 proteins are regarded as the next generation of molecular rapid diagnosis technology [20,21]. The CRISPR/Cas assay is more specific than other nucleic acid tests, such as recombinant polymerase isothermal amplification (RPA) and loop-mediated isothermal amplification (LAMP) [22]. Therefore, scientists have combined PCR, RPA, and LAMP amplification technologies to develop a variety of CRISPR/Cas system-based detection platforms for the prevention and control of various viruses, such as SHERLOCK, DETECTR, HOLMES, and AIOD-CRISPR [23,24,25,26], which display fine performance in rapid, highly sensitive, and accurate viral detection. In this study, we intended to establish a LAMP-CRISPR approach through combining the advantages of LAMP with those of the CRISPR/Cas12a system, thereby achieving sensitive, visual, and point-of-care PCV2 detection. In particular, this method was further evaluated and compared with the traditional qPCR approach in the detection of clinical samples.

## 2. Materials and Methods

### 2.1. Viruses and Clinical Samples

Porcine circovirus 1 (PCV1), porcine circovirus 2 (PCV2), porcine circovirus 3 (PCV3), genomic DNAs of porcine parvovirus (PPV) and porcine pseudorabies virus (PRV), cDNAs derived from porcine reproductive and respiratory disorder syndrome virus (PRRSV), classical swine fever virus (CSFV), and porcine epidemic diarrhea virus (PEDV) positive samples were maintained in the Hunan Provincial Key Laboratory of Veterinary Protein Engineering Vaccine, Hunan Agricultural University. All clinical samples were also provided by the laboratory and stored at −80 °C until use.

### 2.2. Nucleic Acid Preparation 

The viral DNAs or RNAs of PCV1, PCV2, PCV3, PRRSV, PPV, CSFV, and PEDV were extracted using the TIANamp viral DNA/RNA kit (TIANGEN, Beijing, China) according to the manufacturer’s instructions and eluted in nuclease-free water. The viral RNAs were reverse transcribed with M-MLV Reverse Transcriptase (Promega, Beijing, China). All cDNAs and DNAs were stored at −80 °C until use. 

### 2.3. LAMP Primers Design and crRNA Preparation

Twenty-two nucleotide sequences of the PCV2 *rep* gene were aligned using DNAMAN to identify the conserved regions, which were utilized to identify a suitable target sequence for the LAMP assay (marked by a red box) (Figure S1). The LAMP primers were designed using the PrimerExplorer V5 software (http://primerexplorer.jp/e/ accessed on 1 June 2021) in the conserved nucleotide region of the *rep* (Table S1). The crRNAs were designed using the online tool CRISPR-DT targeting the LAMP amplified conserved regions and appended with the T7 promoter sequence (Table S1). The synthesized oligonucleotides were annealed to prepare transcription templates, which were then purified by gel extraction. Using the HiScribe T7 Quick High Yield RNA Synthesis Kit (New England Biolabs, Beijing, China) and phenol chloroform extraction, crRNAs were transcribed and purified according to the manufacturer’s instructions. The crRNAs were stored at −80 °C until use. All primers and the FAM-BHQ1-labeled single-stranded DNA reporter (tz-Cas12aDT) were synthesized by Tsingke (Beijing, China).

### 2.4. Preparation of huLbCas12a

The whole huLbCpf1 fragment, along with 6HisTag, was amplified from the 6His-MBP-TEV-huLbCas12a plasmid (Addgene, Watertown, MA, USA), followed by digestion with *EcoR*I and *Xho*I (New England Biolabs, Beijing, China), and then cloned into the pET-28a vector. Following validation by DNA sequencing (Tsingke, Beijing, China), this pET-28a-6His-huLbCas12a construct was transformed into Rosetta DE3 (Tsingke, Beijing, China), and a single bacterial clone was picked up for small-scale culture. Then, 5 mL overnight culture was added into a 0.5 L self-induced culture medium containing 0.1% kanamycin antibiotic for protein expression at 25 °C for 16 h. Next, the culture was centrifuged at 2400× *g* for 15 min and resuspended in lysis buffer (50 mM KH_2_PO_4_, 10 mM Imidazole, 300 mM Tris-HCl, 50 mM KCl, 300 mM NaCl, pH8.0) with Triton X-100, 0.1 mM PMSF, and 5% β-me. The suspended bacteria were sonicated on ice, followed by centrifugation. The supernatant was then collected and added to Ni-NTA Agarose (QIAGEN, Hilden, Germany) for one hour. Finally, the wash buffers (50 mM KH_2_PO_4_, 300 mM Tris-HCl, 50 mM KCl, 300 mM NaCl, pH8.0) separately containing 10-, 30-, 50-, 100-, and 500-mM Imidazole were used to elute the huLbCas12a protein. The purified huLbCas12a protein was validated by Western Blot with an anti-His antibody (Solarbio, Beijing, China), and then aliquoted and stored at −80 °C until use.

### 2.5. Determination of Target Cleavage Activity of the huLbCas12a Protein

To verify the trans- and cis-cleavage activities of the purified huLbCas12a, the extracted PCV2 genome was used as a template to amplify the *rep* gene fragment by PCR using rep-F and rep-R (Table S1). PCR was conducted on a 2720 Thermal Cycler with a foremost denaturation step at 98 °C for 3 min, followed by 38 cycles at 98 °C for 30 s, 56 °C for 30 s, and 72 °C for 1 min, and terminated with a final extension at 72 °C for 10 min. The size of PCR products was 945 bp, with the targeted position of crRNA3 located at a position of 450 bp. The product was added to the reaction system with the complexes of huLbCas12a and crRNA3, incubated at 37 °C for 30 min, then inactivated at 95 °C for 10 min. The product was then observed by 2% agarose gel electrophoresis. Then, a FAM-BHQ1-labeled ssDNA reporter was added for visual observation under ultraviolet light.

### 2.6. Optimization and Evaluation of the LAMP-CRISPR Detection Method

**Figure 1:**
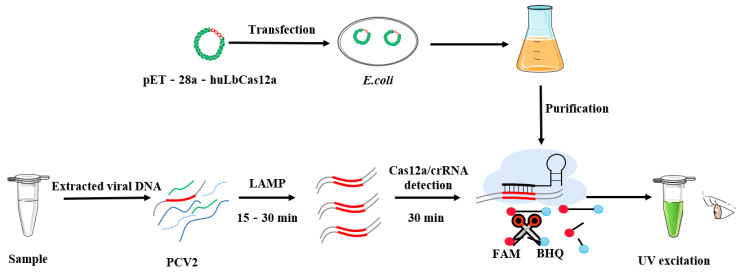
Schematic of LAMP amplification, hubLCas12a protein preparation and Cas12a/crRNA cleavage for visualizing the presence of PCV2-specific nucleic acids.

The LAMP-CRISPR detection procedure includes initial loop-mediated isothermal amplification (LAMP) of the *rep* gene from target samples, followed by purified Cas12a reaction, and result observation (Figure 1). A series of optimizations have been made to the LAMP reaction. The LAMP reaction was conducted using 2.5 μL 10 × LAMP Master Mix, 0.32 U Bst3.0 DNA polymerase (Sangon, Shanghai, China), 2.0 μM inner primer, 0.2 μM outer primer, 0.4 μM loop primer, 2.0 μL DNA template, and 3.5 μL distilled water at 63 °C for 30 min [27].

To achieve the best performance, a series of optimizations were performed for the CRISPR/Cas12a reaction. Briefly, crRNAs of three target sites were screened to induce optimal huLbCas12a shearing activity, and the concentration of huLbCas12a and crRNA was optimized from 250:250 nM to 250:500 nM. The CRISPR/Cas12 reaction system was comprised of 2 μL detection buffer (500 nM NaCl, 100 nM Tris-HCl, 100 mM MgCl_2_, 100 mM BSA, pH7.9), 2 μL LAMP amplification products, 2 μM FAM-BHQ1-labeled ssDNA reporter, and nuclease-free sterilization water up to 20 μL. The fluorescence intensity was measured by ABI QuantStudio 5 (Applied Biosystems, Thermo Fisher Scientific, Waltham, MA, USA) and the tube was observed under a gel imaging system with a UV channel in the 30th min.

### 2.7. LAMP-CRISPR Fluorescence Assay

The PCR-amplified *rep* fragments were digested by *BamH* I and *Kpn* I, and inserted into pUCmT as the standard plasmid. The copies of pUCmT-rep were calculated as follows: Copy number = (Amount × 6.02 × 10^23^)/(DNA length × 10^9^ × 660 Da/bp). The pUCmT-rep was diluted from 1.0 × 10^6^ to 1.0 × 10^0^ copies/μL by a tenfold gradient dilution. The sensitivity of the LAMP-CRISPR detection was assessed with the LAMP products amplified using different copy numbers of pUCmT-rep. To verify the specificity of the LAMP-CRISPR detection, seven porcine viruses, including CSFV, PCV2, PCV1, PCV3, PPV, PEDV, and PRRSV, were tested. The fluorescence intensity was measured by ABI and observed under UV light.

### 2.8. Efficiency Assessment of the LAMP-CRISPR Detection System for Clinical Samples Detection

For the evaluation of clinical samples, qPCR is regarded as the gold standard. Taqman probes and qPCR primers were designed to target the conserved regions of the *cap* gene. The reaction system of qPCR detection contained 10 μL 2 × AceQ real-time PCR Probe Master Mix (Vazyme, Nanjing, China), 400 nM PCV2-*cap*-F/R, 200 nM PCV2-*cap*-P Taqman probe (Table S1), 1 μL DNA template, and an appropriate amount of RNase-free water, which was conducted on ABI QuantStudio 5 with a foremost denaturation step at 95 °C for 5 min, followed by 45 cycles of denaturation at 95 °C for 10 s, and annealing and extension at 60 °C for 30 s. The acquisition of fluorescent signals was recorded at the end of each cycle. After the reaction, the ABI system automatically determined the Ct values. Therefore, the comparison of the detection efficiency of the LAMP-CRISPR detection system and qPCR detection was conducted on 30 clinical samples from 30 diseased pigs collected across different areas. In both approaches, the corresponding fluorescent probe was used, thereby obtaining the coincidence rate between them.

### 2.9. Statistic Analysis

Data were statistically processed by GraphPad Prism 8, *t*-test and one-way ANOVA were used for statistical analysis of the obtained data, where *, *** represent *p* < 0.05, <0.001, respectively.

## 3. Results

### 3.1. Expression and Purification of the huLbCas12a Protein in E. coli

**Figure 2:**
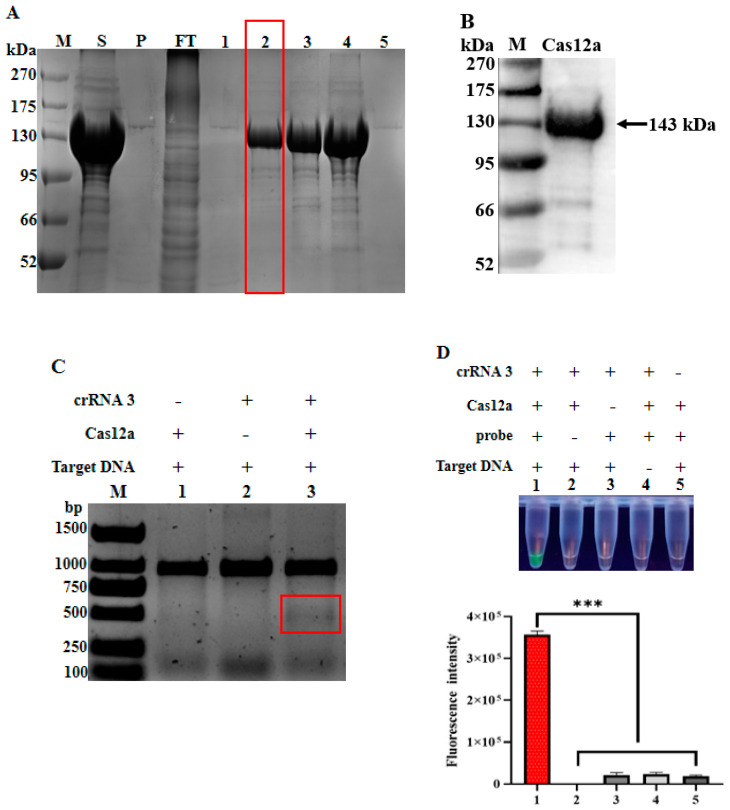
Purification huLbCas12a protein and its cleavage activity analysis. (**A**) The supernatant and precipitation of lysed Rosetta DE3 was fractioned by SDS-PAGE and stained by Coomassie blue. M: PM0500 protein Marker; P: Precipitation; S: Supernatant; FT, Flow-through sample. 1, 2, 3, 4, 5: The proteins eluted by buffers containing different concentrations of imidazole including 10, 30, 50, 100, and 500 mM, respectively. The red-marked frame indicated the LbCas12a protein used for subsequent experiments. (**B**) Western blot analysis of the purified huLbCas12a protein. (**C**) The verification of the cis-cleavage activity of the LbCas12a protein was assessed by its cleavage of target DNA fragment and presented by nucleic acid gel electrophoresis. The red frame marked area represents the cleavage products. M: DL3000 DNA Marker; the presence (+) or absence (−) of individual components is indicated above. (**D**) The trans-cleavage activity of LbCas12a protein. the presence (+) or absence (−) of individual components is indicated above the panel. Corresponding fluorescence intensity of each tube is shown in the lower panel (*n* = 3 technical replicates, value represents mean ± SEM, where *** represents *p* < 0.001).

Following the construction of a gene fragment encoding the 6His-tagged huLbCas12a into the pET-28a vector and a series of expression optimizations, including bacterial strain selection, temperatures, and culture time, the protein of interest was found to be effectively expressed in the *E. coli* Rosetta DE3 strain at the temperature of 25 °C for 16 h without inducer. Then, the bacterial cells were subjected to the sequential procedures of collection, suspension, sonication, and centrifugation. Intriguingly, the Coomassie blue staining result revealed that the His-tagged huLbCas12a protein was mainly present in the supernatant, thereby allowing the large-scale expression and purification of this protein under native conditions. Subsequently, the cell lysates were subjected to Ni-immobilized metal affinity chromatography for the purification of huLbCas12a (Figure 2A). Notably, huLbCas12a proteins were prepared with high purity when the wash buffer contained 30 mM imidazole, as confirmed by Coomassie staining. Additionally, specific huLbCas12a proteins were further validated by Western Blot (Figure 2B).

### 3.2. Target Cleavage Activity of huLbCas12a Protein

Having obtained purified huLbCas12a protein, we next tested whether it has corresponding activities. As described in Materials and Methods, when purified huLbCas12a protein, a 945 bp PCV2 *rep* DNA fragment, and crRNA3 targeting this gene were put together, the activities of Cas12a were observed, as demonstrated by the target cleavage of the rep gene fragment. As shown in Figure 2C, the absence of any individual component failed to cut the DNA fragment, while the presence of all three components, as indicated, led to the cleavage of the 945 bp PCV2 rep fragment into two about 450 bp DNA bands (red box), suggesting that this expressed and purified protein has the expected capability in terms of target DNA cleavage. Likewise, only when FAM-BHQ1-labeled ssDNA reporter was applied to the reaction mixture including all three components, intense green fluorescence was observed under ultraviolet light (Figure 2D). Collectively, the above results demonstrate the feasibility of establishing the Cas12a-based PCV2 detection system in a subsequent effort.

### 3.3. Optimization of the LAMP-CRISPR Detection

**Figure 3:**
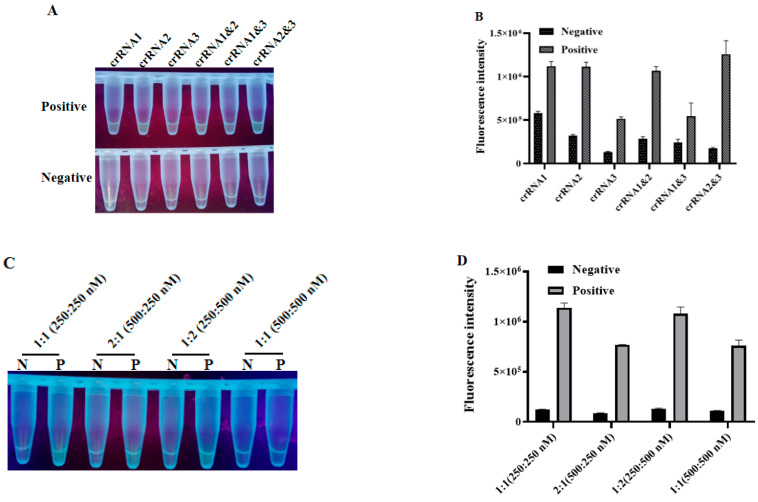
Fluorescence-based optimizations for the CRISPR assay. Different crRNA/huLbCas12a combinations were allowed to cleave target DNA for 30 min. (**A**) Visual results of crRNA screening under UV light. (**B**) Histogram of fluorescence values measured by ABI QuantStudio 5. (**C**,**D**) The optimal ratio of huLbCas12a to crRNA (*n* = 3 technical replicates, value represents mean ± SEM).

The fluorescence CRISPR assay was adopted to screen the best crRNA among the three designed crRNAs and the optimal concentration for huLbCas12a and crRNA. Intriguingly, the crRNA2&3 double targeting group had a higher fluorescence value while presenting a relatively low background fluorescence value, suggesting that they could stimulate strong fluorescence under UV light (Figure 3A), as further confirmed by the quantification of fluorescence strength (Figure 3B). Similarly, the optimized concentrations for the huLbCas12a protein and crRNA were regarded as 250:250 nM since the fluorescence intensity produced by the crRNA/huLbCas12a combination under this concentration reached its highest (Figure 3C). Furthermore, a 1:1 ratio between those two molecules facilitated their binding (Figure 3D).

### 3.4. Sensitivity and Specificity Test of LAMP-CRISPR Detection

**Figure 4:**
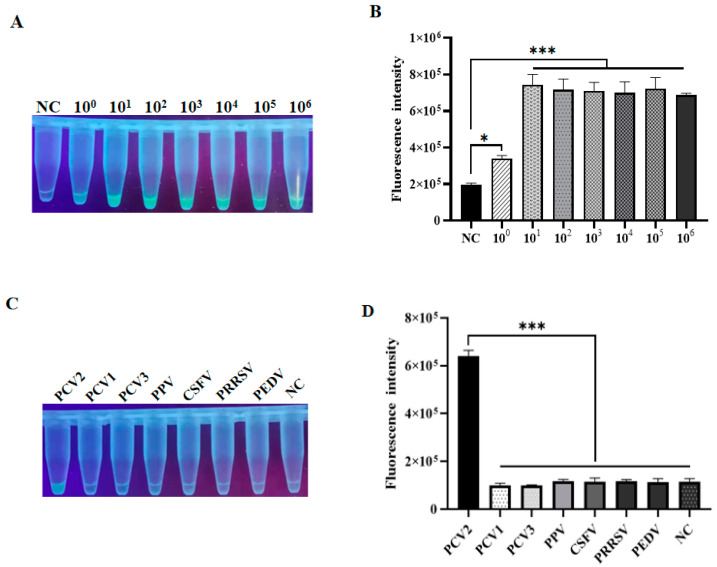
Fluorescence-based analysis of the sensitivity and specificity of LAMP-CRISPR. (**A**) Sensitivity of this method was determined by targeting various concentrations of pUCmT-rep plasmid. (**B**) The end-point fluorescent intensity was measured by ABI QuantStudio 5. (**C**) Visualization by UV light. (**D**) Florescence quantification after performing LAMP-CRISPR assay on DNA viruses individually including PCV1, PCV3, PPV, and PCV2, and RNA viruses like CSFV, PRRSV, and PEDV. NC: Negative control (*n* = 3 technical replicates, value represents mean ± SEM, where *, *** represent *p* < 0. 05, < 0.001, respectively).

To evaluate the sensitivity of the LAMP-CRISPR method, its capability to detect target *rep* DNA inserted into the pUCmT vector, which was serially diluted into various copy numbers, was assessed as described above. Interestingly, the test result demonstrated that the fluorescence intensities of 1.0 × 10^6^ to 1.0 × 10^1^ copied/μL plasmids were almost similar, while reaching their lowest when the plasmid copy number was equal to 1 × 10^0^ copies/μL, which was almost the limit for visual observation under UV light, as compared with negative control (Figure 4A), suggesting that the detection limit for this method could be regarded as 1.0 × 10^0^ copied/μL, as also consistent with fluorescence analysis by ABI QuantStudio 5 (Figure 4B). Considering the fact that mixed infections are very common, especially co-infection of polytype porcine circovirus in actual clinical samples with porcine diseases, the specificity of the LAMP-CRISPR method was further evaluated. Intriguingly, upon the individual presence of different viral genomic DNAs, this approach could accurately and specifically distinguish PCV2 from other viruses, including PCV1, PCV3, PPV, PCV2, CSFV, PRRSV, CSFV, and PEDV (Figure 4C), as indicated by significant differences in fluorescence intensities among these viruses (Figure 4D). Collectively, these data robustly demonstrate the fine sensitivity and specificity of this LAMP-CRISPR detection method.

### 3.5. Comparation of LAMP-CRISPR and Taqman-Based qPCR in Detecting Clinical Samples

**Figure 5:**
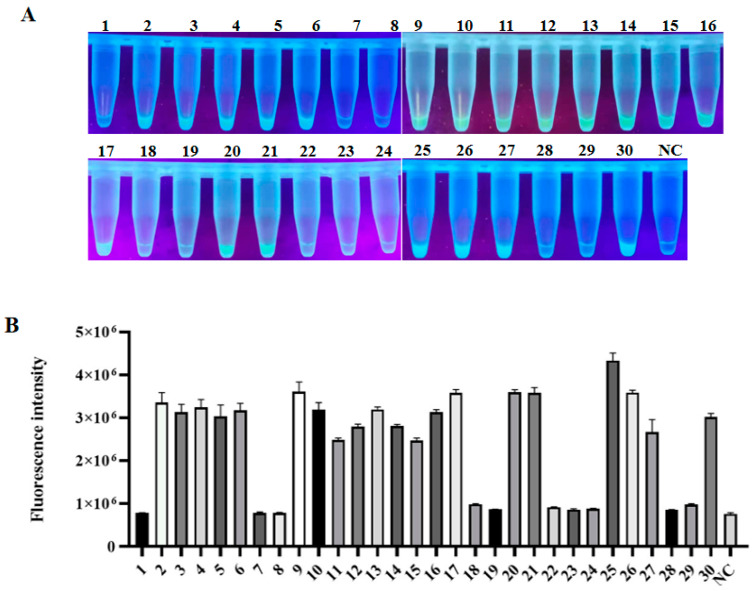
Detection of PCV2 in swine clinical samples by LAMP-CRISPR approach. (**A**) 30 clinical blood samples were tested by the LAMP-CRISPR assay. (**B**) Fluorescence intensity corresponding to each indicated tube. (*n* = 3 technical replicates, value represents mean ± SEM).

Finally, the reliability of LAMP-CRISPR established in this study was further confirmed by comparing it with the frequently used Taqman-based qPCR in detecting 30 clinical blood samples. As shown in Table 1, qPCR revealed that 66.6% (20/30) of samples presented PCV2 positive. Likewise, LAMP-CRISPR showed that 20 samples were PCV2 positive, presented by strong fluorescent signals, while the other 10 samples were negative, presented by very low or background signals (Figure 5A,B). This result was completely consistent with that of qPCR detection. Notably, some low-copy PCV2 clinical samples detected by qPCR could still be captured by the LAMP-CRISPR, further verifying the sensitivity and specificity of this detection method.

## 4. Discussion

Since the 1990s, PCV2 has become one of the major economical pathogens in the pig industry worldwide. Although PCV2 is a small DNA virus, it has been considered to be quite conserved until reported isolates of PCV2 showed significant genetic variations [28]. Given the increase in PCV2 variants and co-infection with other swine viruses and the lack of laboratory facilities for diagnosis in different scale farms, diagnosis of PCV2 often requires the transportation of infected samples to qualified laboratories, and this process may delay the diagnosis and increase the risk of transmission. Though PCR and qPCR can detect PCV2 nucleic acids with high sensitivity [14,15], the requirements for expensive equipment and specialized personnel often limit diagnostic applications in poorly equipped laboratories or in the field, especially in developing countries. Similarly, antigen detection assays, such as ELISA and IFA, are not so reliable due to their low sensitivity and susceptibility to contamination [13,18]. Therefore, a portable, visual, and real-time detection method, such as LAMP coupled with CRISPR established in this study, which is able to efficiently identify the presence of PCV2 nucleic acids, would greatly facilitate the diagnosis of this pathogen and the control of corresponding diseases.

Initially, we provided an optimal protocol for the expression of the huLbCas2a protein in a prokaryotic expression system, including some important parameters such as bacterial strain, temperature, time, and an optimal buffer to elute purified protein using nickel-fixed metal affinity chromatography. These efforts not only greatly reduced the cost of PCV2 detection but also provided a useful reference for the field. More importantly, this recombinant HuLbCas12a protein had desirable activity. Notably, compared with other efforts [29], our protocol not only obtained a desirable target protein amount but also reduced the step of removing a tag, such as MBP or SUMO, which is usually adopted to favor protein solubility. Additionally, a requirement for IPTG induction was also omitted [30].

Subsequently, we intend to establish a LAMP coupled with a CRISPR/Cas12a assay to achieve real-time field detection of PCV2. In contrast to a previously established method, which adopted the Taqman-based qPCR assay to detect PCV2 by designing primers and probes targeting the ORF2 encoding the cap protein [15], we designed LAMP primers and crRNAs targeting the conserved region of the ORF1 gene encoding the Rep protein after the alignment and analysis of 22 PCV2 *rep* gene sequences. More importantly, this LAMP-CRISPR displayed a detection limit of 1 copy/μL, in contrast to the Taqman-based qPCR approach, which has a sensitivity of 100 copies/μL [15]. Furthermore, the former also demonstrated a fine specificity since it specifically detected PCV2 only, rather than other swine viruses. 

It was worth noting that LAMP-CRISPR-based viral detection was more efficient and prominent, especially in practical application. First of all, FAM-BHQ1, as a fluorescence reporter, has ultraviolet transillumination and facilitates real-time visualization of results, making it easier to capture. Second, although the efficiency of huLbCas12a cleavage may be variable and subject to the influence of some factors such as pH and sample complexity, the high-efficiency amplification of LAMP can compensate for the reduced efficiency of huLbCas12a. Thus, while the general efficiency of the LAMP-CRISPR method may not be compromised, satisfactory reactivity is still attained. Notably, the detection result can be obtained within one hour, thereby allowing the acquisition of precious time for the epidemic prevention and clinical treatment of PMWS. Third, the crRNAs targeting the PCV2 *rep* may stimulate huLBCas12a activity, making the fluorescence signals reach their highest intensity. Similarly, a previous study has demonstrated that the fine combination of crRNAs can improve the detection sensitivity of Cas13a by activating more Cas13a per target RNA [31]. In addition, the use of multiple crRNAs targeting different regions of a gene also protects against a potential loss of detection due to naturally occurring viral mutations. The crRNA2&3 double target combination in this study not only displayed an appreciated efficiency but also partially confirmed these previous views. Finally, the established LAMP-CRISPR PCV2 detection could be conducted in a thermostatic water bath, thereby allowing it to be applied in underequipped laboratories or in the field and breaking the limitations of traditional detection methods. 

Last but not least, this LAMP-CRISPR method was further validated with 30 clinical blood samples. Its accuracy was completely consistent with that of qPCR, suggesting that its reliability was comparable to that of the current gold standard. Additionally, the LAMP-CRISPR assay requires less time than qPCR. Moreover, the detection process was simpler. An effort to develop a PCV2 detection kit based on this LAMP-CRISPR method established in this study is ongoing.

## 5. Conclusions

In conclusion, a visual, rapid, low-cost, sensitive, specific, and portable nucleic acid detection method for PCV2 was established through coupling LAMP with CRISPR/Cas12a. The advantages and properties of this LAMP-CRISPR approach demonstrate its great potential in the field detection of PCV2, thereby offering an effective way to monitor PCV2 in time and prevent the occurrence and transmission of PCV2 at an early stage.

## 6. Patents

A.W., L.T., F.L., L.L., D.D., Y.Y., Z.Y., N.W., H.L., Z.D., Y.W. and Y.H. have submitted a patent application on this technology with an application No. (2022107276727, China).

## Figures and Tables

**Table 1:** The results revealed by qPCR in detecting PCV2 among clinical samples. The Ct value represents the threshold cycle value.

**Simple Number**	**1**	**2**	**3**	**4**	**5**	**6**	**7**	**8**	**9**	**10**
**Ct value**	-	33.82	23.54	31.55	32.84	32.13	-	-	25.59	35.44
**Simple number**	**11**	**12**	**13**	**14**	**15**	**16**	**17**	**18**	**19**	**20**
**Ct value**	35.28	31.77	28.44	34.38	35.80	28.39	20.15	-	-	25.81
**Simple number**	**21**	**22**	**23**	**24**	**25**	**26**	**27**	**28**	**29**	**30**
**Ct value**	25.35	-	-	-	17.14	31.19	35.02	-	-	32.71

## Data Availability

The data presented in this study are available in the Supplementary Materials of this manuscript.

## Supplementary Materials

The following supporting information can be downloaded at: https://www.mdpi.com/article/10.3390/ani12182413/s1, Figure S1: Sequence alignment of PCV2 *rep* genes; Table S1: Sequence information of LbCas12a-F/R, crRNAs, ssDNA reporter, LAMP primers, rep-F/R, qPCR primers and Taqman probe used in this study.
