# Supplementary material for: LAMP Coupled CRISPR-Cas12a Module for Rapid, Sensitive and Visual Detection of Porcine Circovirus 2
Source: Animals (Basel). 2022 Sep 14;12(18):2413. doi: 10.3390/ani12182413 (PMC9495112; doi:10.3390/ani12182413)
Supplement: Supplementary file 1 [file animals-12-02413-s001.zip › animals-1865589-supplementary/Supplementary files/Table S1.pdf]

Table S1. Sequence information of LbCas12a-F/R, crRNAs, ssDNA reporter, LAMP primers and rep-F/R used in this study.

Name	Sequences（5'→3'）
LbCas12a-F	GCGGAATTCCATCATCACCATCACCACATGAGCAAGCTGGAGAAG
LbCas12a-R	CCGCTCGAGGGATCCCTTTTTCTTTTTTGCCTGGC
crRNA1-F	TAATACGACTCACTATAGG AATTTCTACTAAGTGTAGAT AAAAGTTCAGCCAGCCCGCG
crRNA1-R	CGCGGGCTGGCTGAACTTTTATCTACACTTAGTAGAAA TTCCTATAGTGAGTCGTATTA
crRNA2-F	TAATACGACTCACTATAGG AATTTCTACTAAGTGTAGAT TCAGAAATTTCCGCGGGCTG
crRNA2-R	CAGCCCGCGGAAATTTCTGAATCTACACTTAGTAGAAA TTCCTATAGTGAGTCGTATTA
crRNA3-F	TAATACGACTCACTATAGG AATTTCTACTAAGTGTAGAT CGCGGGCTGGCTGAACTTTT
crRNA3-R	AAAAGTTCAGCCAGCCCGCGATCTACACTTAGTAGAAA TTCCTATAGTGAGTCGTATTA
tz-Cas12aDT	(FAM)CCGGAAAAAAAAAAAACCGG(BHQ1)
F3	GGGAGTCTGGTGACCGTT
B3	GGTGGTTTCCAGTATGTGGT
FIP	ACGCTTCTGCATTTTCCCGCTC-GAGCAGCACCCTGTAACG
BIP	ATGTACACGTCATTGTGGGGCC-CCGGGTCTGCAAAATTAGCA
LF	TTCAAAAGTTCAGCCAGCCC
LB	ACCTGGGTGTGGTAAAAGCA
rep-F	CGGGGTACCATGCCCAGCAAAAAGAA
rep-R	CGGGGTACCTCAGTAATTTATTTCATATGG
