# Supplementary figures and images for: LAMP Coupled CRISPR-Cas12a Module for Rapid, Sensitive and Visual Detection of Porcine Circovirus 2
Source: Animals (Basel). 2022 Sep 14;12(18):2413. doi: 10.3390/ani12182413 (PMC9495112; doi:10.3390/ani12182413)

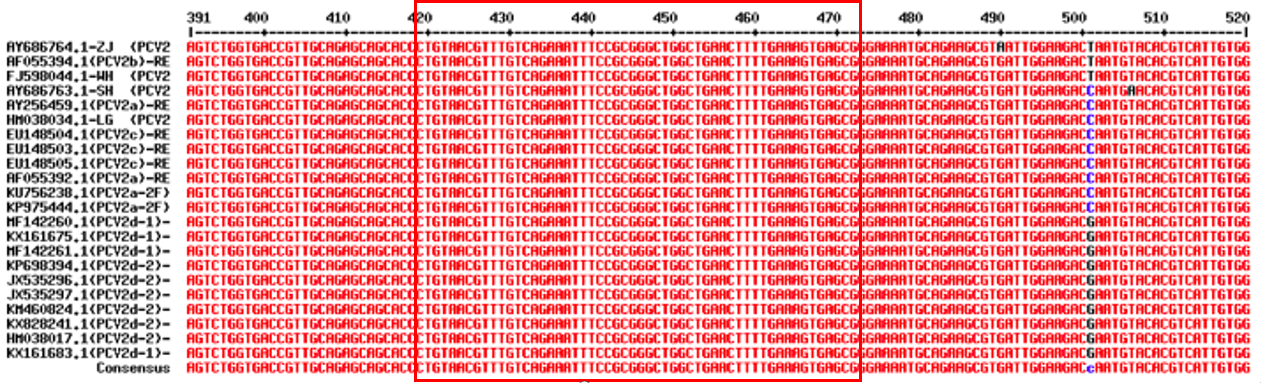

Supplement: Supplementary file 1 [file animals-12-02413-s001.zip › animals-1865589-supplementary/Supplementary files/Figure 1S.tif]

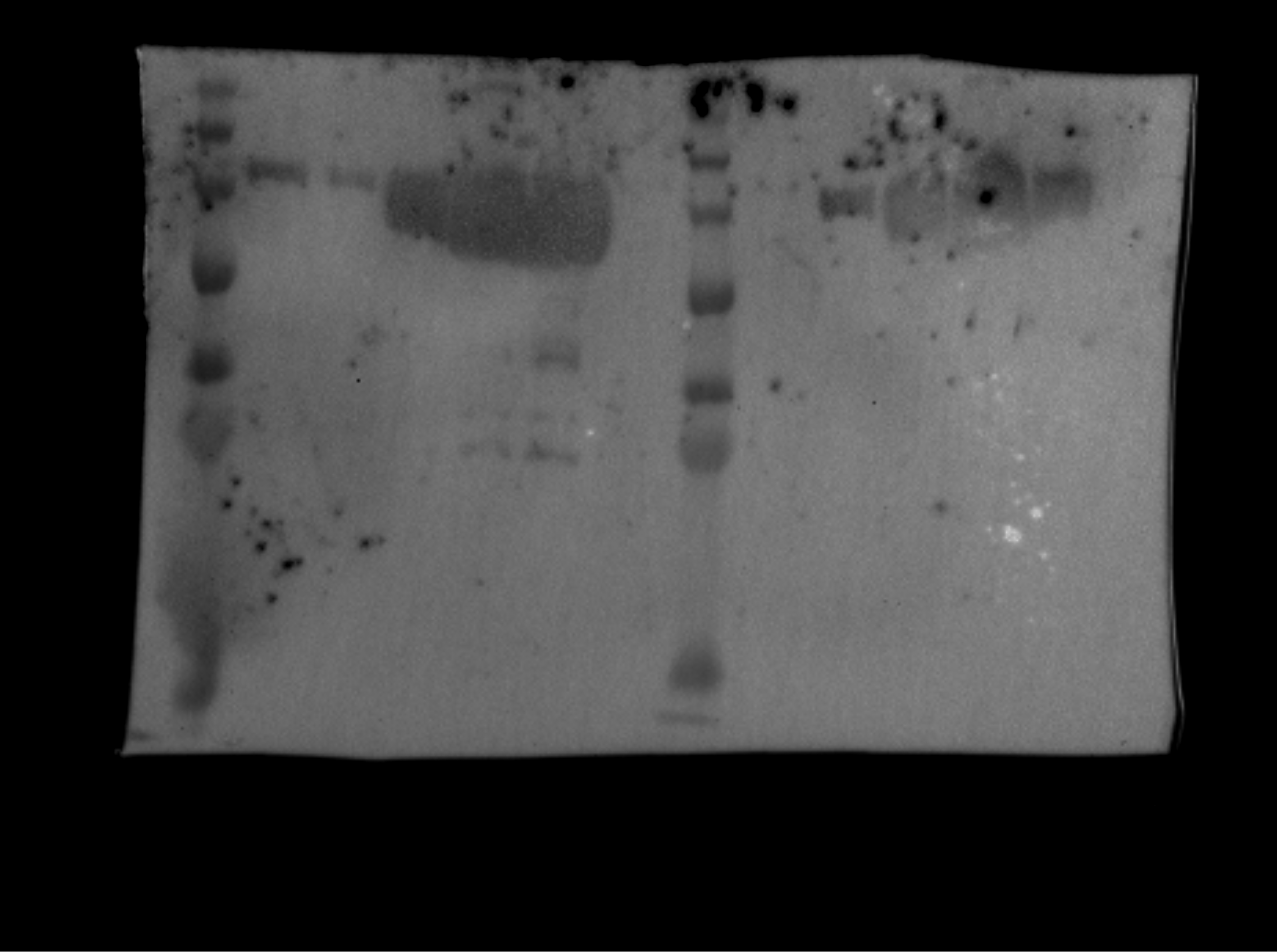

Supplement: Supplementary file 1 [file animals-12-02413-s001.zip › animals-1865589-supplementary/Supplementary files/Figure S2.tif]
